# DMRT Transcription Factors in the Control of Nervous System Sexual Differentiation

**DOI:** 10.3389/fnana.2022.937596

**Published:** 2022-07-26

**Authors:** Rafael Casado-Navarro, Esther Serrano-Saiz

**Affiliations:** Tissue and Organ Homeostasis Program, Centro de Biología Molecular Severo Ochoa (CSIC-UAM), Madrid, Spain

**Keywords:** *Dmrt*, sexual differentiation, nervous system, *doublesex*, *dmd*, conservation

## Abstract

Sexual phenotypic differences in the nervous system are one of the most prevalent features across the animal kingdom. The molecular mechanisms responsible for sexual dimorphism throughout metazoan nervous systems are extremely diverse, ranging from intrinsic cell autonomous mechanisms to gonad-dependent endocrine control of sexual traits, or even extrinsic environmental cues. In recent years, the DMRT ancient family of transcription factors has emerged as being central in the development of sex-specific differentiation in all animals in which they have been studied. In this review, we provide an overview of the function of *Dmrt* genes in nervous system sexual regulation from an evolutionary perspective.

## Introduction

The brain of most animals displays sexual differences at the level of neuronal number, gene expression, synaptic connectivity, and behavior (Trabzuni et al., 2013; Yang and Shah, 2016; McCarthy, 2020; Goodwin and Hobert, 2021). These sexual differences impact innate behaviors, those displayed without any prior learning or training, and as a consequence, innate behaviors have a strong sex bias. More relevant to human pathology, the study of sexual differentiation of the brain is of major significance based on the premise that it might uncover factors involved in the general etiology and sexual skewing of neuropsychiatric disorders (McCarthy, 2016; Zagni et al., 2016).

The molecular mechanisms that determine the sex of an organism are almost as diverse as the species themselves. However, among this wide variety, *Doublesex* and *mab-3* related transcription factors (*Dmrt*) are unique in their conservation, in organisms as diverse as mammals, fishes, birds, frogs, insects, and nematodes. Their conserved role in gonadal differentiation has been discussed in excellent reviews elsewhere (Matson and Zarkower, 2012; Bellefroid et al., 2013). In this review, we consider the conserved function of *Dmrt* genes in nervous system sexual differentiation. We comprehensively describe studies in two invertebrate genetic model organisms, *Drosophila melanogaster* and *Caenorhabditis elegans*, which have been studied with unprecedented detail. Male and female connectomes are fully reconstructed in *C. elegans* (White et al., 1986; Cook et al., 2019) and in the *Drosophila* female (Zheng et al., 2018; Scheffer et al., 2020), and work over the years has revealed several sexual dimorphisms (Goodwin and Hobert, 2021). High genetic access, complemented with stereotyped sex-specific behaviors, has allowed for a comprehensive study of *Dmrt* genes in sexual differentiation of the nervous system of *C. elegans* and *Drosophila*. The first evidence of a *Dmrt* gene controlling nervous system sexual differentiation was published in 1980, and was related to *Drosophila's* peripheral nervous system (Baker and Ridge, 1980). The authors found that *doublesex* (*dsx)* was required for differentiation of sex combs, a male-specific array of modified mechanosensory bristles needed for mating (Spieth et al., 1952; Cook, 1977; Ng and Kopp, 2008). Likewise, *mab-3* was shown to be involved in male-specific sensory organ development and behavior in *C. elegans* (Shen and Hodgkin, 1988; Yi et al., 2000). However, more than 30 years have passed since these seminal initial works (Baker and Ridge, 1980; Shen and Hodgkin, 1988), and no systematic research in other organisms has been conducted. In the present work, we collect and summarize relevant data about the sexual regulation of the nervous system by the *Dmrt* genes *dsx* and *dmds* (*Dmrt* nomenclature in *C. elegans*), as a roadmap for studying the potential conserved function of *Dmrt* genes in the sexual regulation of the nervous system of other organisms. In addition, we catalog all those animal species in which *Dmrt* expression in neural tissues has been demonstrated, evidencing *Dmrt* function, and highlighting its involvement in sex-specific traits (where present) (Table 1).

**Table 1:**
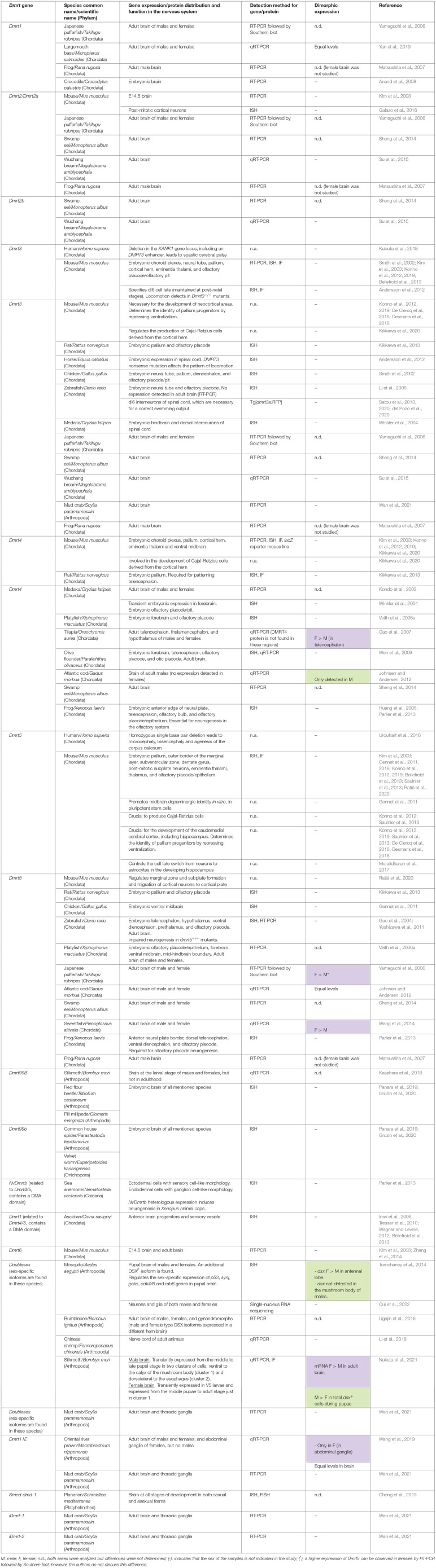
*Dmrt* gene expression and function in the nervous system of diverse metazoan groups.

M, male; F, female; n.d., both sexes were analyzed but differences were not determined; (-), indicates that the sex of the samples is not indicated in the study; (^*^), a higher expression of Dmrt5 can be observed in females by RT-PCR followed by Southern blot, however, the authors do not discuss this difference.

## Brief Description of the *Dmrt* Gene Family

*Dmrt* genes are defined by a DNA-binding region called the DM domain. This motif was named after the founding members *dsx* in *Drosophila* and *mab-3* in *C. elegans*. Both genes encode transcription factors that perform analogous functions in sexual development. Interestingly, *dsx* (in its male isoform, see below) can partially substitute for *mab-3 in vivo* in the peripheral nervous system of *C. elegans* (Raymond and Zarkower, 1998). The DM domain encodes an intertwined zinc-finger DNA-binding motif, followed by an alpha-helical recognition domain that confers sequence-specific DNA binding through a 13 bp pseudo-palindromic DNA motif (Murphy et al., 2007, 2015). Outside the DM region, *Dmrt* genes do not share much homology, except for the DMA subfamily (*Dmrt3, Dmrt4, and Dmrt5* in mammals, *dmrt93B* and *dmrt99B* in *Drosophila*, or *dmd-4* in *C. elegans*). Although the role of the DMA domain is unclear, recent evidence in *C. elegans* points to sex-specific protein stabilization through ubiquitin binding to the DMA domain. The human DMRT3 DMA domain is also able to bind ubiquitin, at least *in vitro*, and when the full protein was ectopically expressed in worm neurons *in vivo*, it was degraded like the worm DMD-4, suggesting conserved molecular functioning of the DMA domain (Bayer et al., 2020).

DMRT proteins regulate the transcription of target genes by activation or repression. They can form heterodimers on DNA *in vitro* and *in vivo*, raising the possibility that they may interact functionally by forming mixed complexes (Murphy et al., 2007). As far as we are aware, most studies on mechanistic aspects of DMRTs derive from DMRT1 function in the mouse gonad, or DSX in *Drosophila*. DMRT1 functionally collaborates with another key male sex regulator, SOX9. Chromatin immunoprecipitation followed by sequencing (ChIP-Seq) experiments show that they both bind to close sites on target genes, suggesting that they have joint effects on target gene expression to maintain and reprogram sexual cell fate (Rahmoun et al., 2017). Related to this function, DMRT1 appears to act as a pioneer transcription factor, binding “closed” chromatin and opening it to allow binding by other regulators such as SOX9, (Lindeman et al., 2021). Lastly, DMRT1 can bind DNA in a highly unusual type of interaction and can bind with different stoichiometries. However, it is not clear what the functional outcome of different stoichiometry might be. In *Drosophila, dsx* acts downstream of sex determination cues, cell autonomously establishing most somatic dimorphisms in distinct tissues. *dsx* is sex-specifically spliced into DSX^F^ and DSX^M^ isoforms. The two proteins share a common region, containing the DM DNA-binding domain and dimerization domains, but differ on sex-specific C-terminal regions (Burtis and Baker, 1989; Erdman and Burtis, 1993; An et al., 1996; Cho and Wensink, 1996; Erdman et al., 1996). These two transcription factors bind largely the same target genes in different tissues in both sexes, but their regulation would depend on the combinatorial activity of other gene-specific factors cooperating with DSX binding (Clough et al., 2014), although such combinatorial action has not yet been described. DSX target genes are involved in signaling (e.g., WNT, ecdysone), transcription and epigenetic regulation (e.g., *bric a brac, Drop, grappa)*, and sex-determination (e.g., *fru, Sxl*, and *dsx* itself). Additionally, DSX cross-regulates known transcription factors involved in *dsx* expression such as *Sex comb reduced* and *Abdominal-B* (Williams et al., 2008; Chatterjee et al., 2011; Tanaka et al., 2011; Clough et al., 2014; Neville et al., 2014).

In somatic tissues, *Dmrt* genes function as developmental regulators that integrate information about sex, position/space, and time, to direct cell populations toward male or female features. This role is perfectly illustrated by the *Drosophila* sex comb—a strictly male-specific organ. In this case, spatial control is accomplished by HOX genes, and sex combs only develop on the first pair of legs, whereas the sex-specificity is controlled by alternative *dsx* splicing. Transcription of *dsx* is induced in the first leg by the HOX gene *Sex combs reduced*, which is not expressed in the more posterior segments (Tanaka et al., 2011). Similarly, in worms, sexual specificity is controlled by the master regulator TRA-1, and temporal specificity by heterochronic pathway genes. As a consequence, several *Dmrt* genes (for which sex-specific functions have been found) are expressed in a male-specific manner, but only from the fourth larval stage, when worms become sexually mature. In some cases, it has been shown that space information is assured by neuron-type-specific regulators of neuronal identity, termed terminal selectors (Hobert and Kratsios, 2019). For instance, *mab-3* expression in SMD motor neurons is controlled by the SMD identity regulator *unc-42*, a homeobox gene (Pereira et al., 2019; Berghoff et al., 2021). In this way, the mechanisms that couple developmental timing to sexual maturation are themselves tightly coupled.

**Figure 1:**
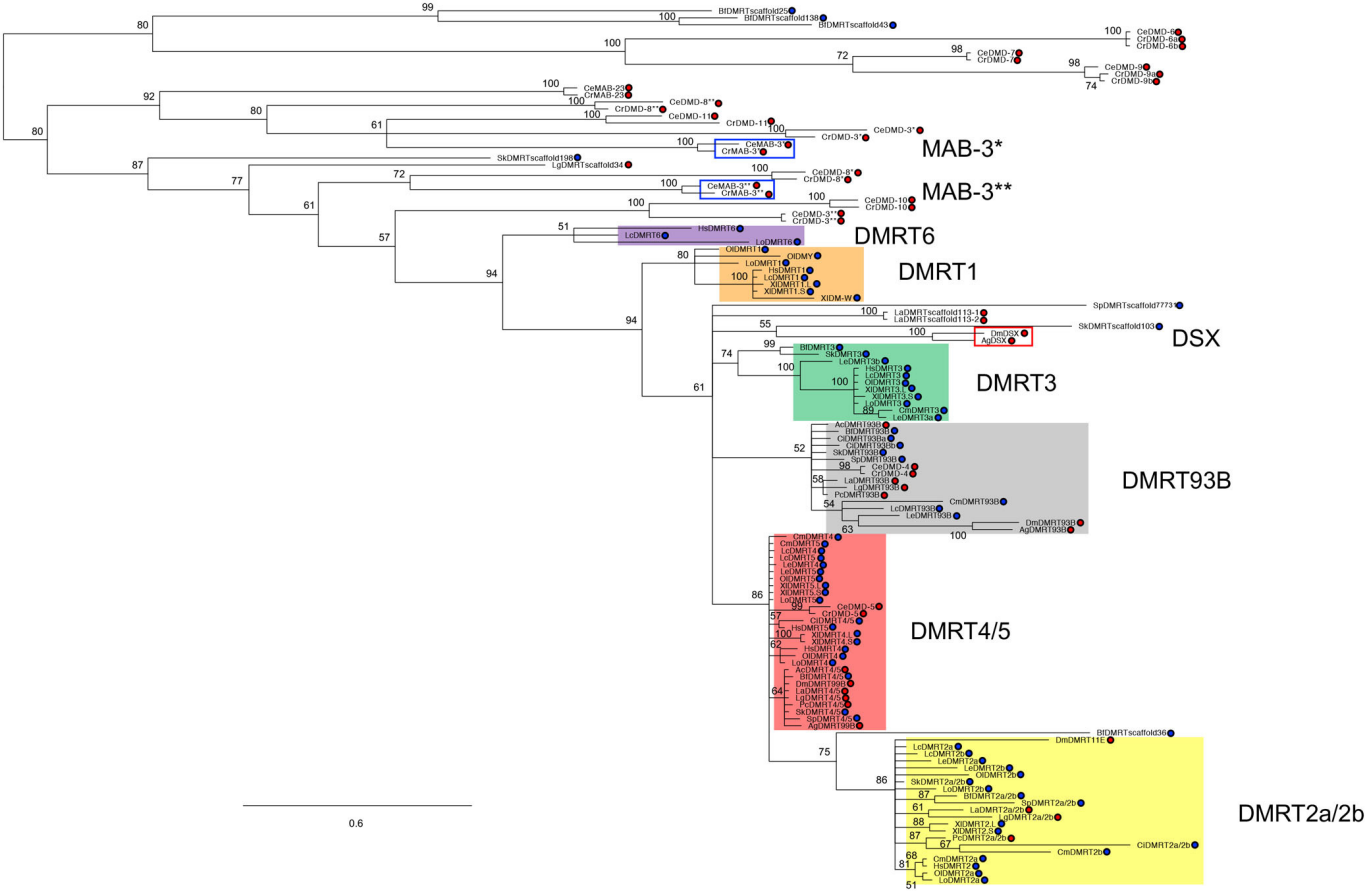
DMRT family phylogeny. Figure adapted from Mawaribuchi et al. (2019). Bayesian tree of bilaterian *Dmrt* family genes. The tree was constructed using protein sequences of the DM domains from 19 species representing eight different phyla in bilateria. Blue and red circles represent Deuterostomia and Protostomia, respectively. * and ** indicate DM domain regions on 5′ and 3′ sides, respectively. *Lingula anatina* (La); *Aplysia californica* (Ac); *L. gigantea* (Lg); *Priapulus caudatus* (Pc); *Caenorhabditis elegans* (Ce); *Caenorhabditis remanei* (Cr); *Anopheles gambiae* (Ag); *Drosophila melanogaster* (Dm); *Saccoglossus kowalevskii* (Sk); *Strongylocentrotus purpuratus* (Sp); *Ciona intestinalis* (Ci); *Branchiostoma floridae* (Bf); *Chondrichthyes, C. milii* (Cm); *L. erinacea* (Le); *L. oculatus* (Lo); *O. latipes* (Ol); *L. chalumnae* (Lc); *Xenopus laevis* (Xl); *Homo sapiens* (Hs).

Most animal genomes contain multiple *Dmrt* genes (Figure 1). The short length of the DM domain (~70 amino acids) hampers the construction of phylogenetic relationships among *Dmrt* paralogs, which is fundamental to unravelling the ancestral developmental functions of this gene family. However, based on several studies that combine syntenic and phylogenic approaches (Ottolenghi et al., 2002; Bellefroid et al., 2013; Wexler et al., 2014; Mawaribuchi et al., 2019) the *Dmrt* gene family has been classified into eight major subsets: *Dmrt1, Dmrt2a/2b (dmrt2, dmrt2a* and *dmrt2b), Dmrt3, Dmrt4/5 (Dmrt4, Dmrt5* and *dmrt99B), Dmrt6, Dmrt7, Dmrt8*, and *dmrt93B* (Figure 1). For instance, in arthropods' genomes there are four *Dmrt* genes (*dsx, dmrt93B, dmrt99B, dmrt11E*), while in *C. elegans* there are ten (*mab-3, mab-23*, and *dmd-3* to *dmd-10*). In mammals there are seven *Dmrt* genes, *Dmrt1* to *Dmrt7* (Ottolenghi et al., 2002; Volff et al., 2003; Bellefroid et al., 2013), and up to three *Dmrt*-like genes (*Dmrt8.1, Dmrt8.2* and *Dmrt8.3*) that lack a functional DM domain but which are clustered in the X chromosome (Veith et al., 2006b). However, in contrast to all other known DMRT proteins, the DM domain-encoding sequence of DMRT8 is not conserved in humans and mice.

For many years, *Dmrt1, mab-3*, and *dsx* have been postulated as orthologues (Ottolenghi et al., 2002; Clough et al., 2014). However, recent research suggests an independent evolution of *Dmrt1, dsx*, and *mab-3* for sex determination and primary sex differentiation (Wexler et al., 2014; Mawaribuchi et al., 2019) (Figure 1). But, regardless of the independent evolution of *Dmrt1* homologues, it seems likely that sexual differentiation regulation is an ancestral function of this gene family (Kopp, 2012; Zarkower and Murphy, 2021). Accordingly, *Dmrt* genes regulate sexual differentiation in species as divergent as nematodes, flatworms, insects, crustaceans, and vertebrates (Matson and Zarkower, 2012). And remarkably, in most species, *Dmrt* genes have been detected in somatic tissues such as the central nervous system, the olfactory placode, as well as in somites (see Table 1 and discussion below). However, their roles in the sexual differentiation of somatic tissues, including the nervous system, are best understood in invertebrate model organisms like *Drosophila* and *C. elegans*, where sex determination is largely cell autonomous.

## *dmd* Genes in the Control of Nervous System Sexual Differentiation in *C. elegans*

In *C. elegans, Dmrt* genes determine sex-specific neuronal traits, which can be grouped into three major categories in the context of distinct and complementary ways to generate sex-specific circuits: control of cell numbers, cell identity, and connectivity, which are eventually reflected in sexually dimorphic behaviors (recently reviewed in Serrano-Saiz and Isogai, 2021).

*C. elegans* has two sexes, males and hermaphrodites (which can be considered somatic females capable of producing sperm). The nervous system of males and hermaphrodites is divided into 294 sex-shared neurons (found in both sexes), while each sex additionally contains sex-specific neurons: eight in hermaphrodites (HSN and VCs), involved in egg-laying behavior, and 93 neurons in males, a few of them located in the head and the ventral nerve cord, with most of them in the tail (Sulston et al., 1980; Sulston, 1983; White et al., 1986; Jarrell et al., 2012; Sammut et al., 2015; Cook et al., 2019). As in the hermaphrodite, these male-specific neurons make extensive connections with sex-shared neurons (Cook et al., 2019).

Sex differences in the nervous system of *C. elegans* occur at multiple levels: sex-specific cell death, sex-specific neurogenesis, and trans-differentiation of glial cells into neurons (Sammut et al., 2015) that lead to sex-specific neuronal numbers. Among the sex-shared neurons (generated in both sexes, positioned in the same manner, and grossly anatomically very similar), sexual differences also appear during sexual maturation at the level of synaptic connectivity across the entire connectome (Cook et al., 2019). Moreover, the systematic mapping of a core identity feature of neurons, their neurotransmitter identity, revealed a number of sexual dimorphisms in neurotransmitter usage as well (Serrano-Saiz et al., 2017b), including sex-specific neurotransmitter switches, and transcriptional scaling. In some cases, these dimorphic features are accompanied by differences in morphology [projections or branching; reviewed by Kim and Kim in this topic (Kim and Kim, 2022)].

The primary sex-determining cue in *C. elegans* is the ratio of X chromosomes to autosomal chromosomes that converges on a terminal “master regulator” of somatic sexual state, the transcription factor TRA-1. When the ratio is high in XX female embryos, levels of TRA-1 are high, and when the ratio is low in XO male embryos, TRA-1 levels are low. TRA-1 acts cell autonomously to regulate an array of intermediate factors that control more specific aspects of sex-specific development and physiology. In a few cases, TRA-1 has been shown to directly control the expression of effector genes [i.e., the repression of the cell death regulator *egl-1* to allow for the survival of HSN hermaphrodite neurons (Conradt and Horvitz, 1999) or the expression of *unc-6/netrin*, implicated in synaptic development to promote male-specific synaptic connectivity (Weinberg et al., 2018)]. However, many TRA-1 effects are controlled by intermediary factors, the most prominent type being the *Dmrt* genes. There are 10 *Dmrt* genes in *C. elegans*, including the founding member of the family, *mab-3*. Six of them are dimorphically expressed (*mab-3, mab-23, dmd-3, dmd-4, dmd-5*, and *dmd-10*). Most of them are expressed in a male-specific manner in male- and sex-shared neurons. In all cases, *Dmrt* male-specific expression occurs as a consequence of *tra-1* low levels. *dmd-4* is an exception, and is degraded in shared neurons in the male tail (Bayer et al., 2020). *dmd*-4 expression in hermaphrodites also depends on *tra-1*, however, through a post-transcriptional regulatory mechanism that involves the stabilization of DMD-4 protein. *dmd-4* is the only *Dmrt* across the *C. elegans* genome that contains a DMA domain, and Bayer et al. described it as a putative ubiquitin-binding domain. The expression of the six *Dmrt* genes does not cover the entire male-specific nervous system, although their expression pattern is not yet complete, which suggests that other factors might be operating downstream of *tra-1*. This is the case for another type of transcription factor, the conserved Zn-finger *lin-29*, which has been found to be expressed in many sex-shared neurons in males (Pereira et al., 2019). Recently, endogenously tagged *gfp* reporters for *mab-3* and *dmd-3* have been generated (Pereira et al., 2019). These reagents have served to clarify the molecular mechanisms that position DMRTs at the right moment, at the right site. *mab-3* and *dmd-3* are precisely timed to begin their expression at the L4 stage when LIN-41, an RNA-binding post-transcriptional regulator, and a key target of the *let-7* miRNA, represses their mRNA (Aeschimann et al., 2017). Moreover, *mab-3* and *dmd-3* are controlled by terminal selectors to be specifically expressed in SMD and PHC neurons, respectively. Finally, the male-specificity of *mab-3* and *dmd-3* expression in the nervous system is controlled by the TRA-1 transcription factor (Pereira et al., 2019).

Importantly, all sexually dimorphic *Dmrt* family members have been implicated in controlling sexually dimorphic neuronal identity features. *mab-3* is expressed in male-specific and sex-shared neurons. It promotes neurogenesis of male-specific sensory neurons by controlling the expression of a bHLH (basic helix-loop helix) transcription factor. During L3, the atonal-class bHLH transcription factor LIN-32 is activated male-specifically in nine pairs of male-specific ray precursor cells (Zhao and Emmons, 1995). *lin-32* expression requires the HOX genes *mab-5* and *egl-5* (Zhao and Emmons, 1995), as well as *mab-3*, which indirectly activates *lin-32* by repressing its repressor *ref-1* (Shen and Hodgkin, 1988; Yi et al., 2000; Ross et al., 2005). In addition to *mab-3*, both *dmd-*3 and *mab-23* control terminal differentiation programs of male-specific ray neurons, such as their neurotransmitter identities (Yi et al., 2000; Lints and Emmons, 2002; Siehr et al., 2011).

*Dmrt* genes are also involved in controlling sexually dimorphic neuronal identity features in sex-shared neurons such as projection morphology, gene expression, and dimorphic connectivity, which ultimately impact function. *mab-3* is expressed in sex-shared embryonically generated ADF and SMD head motor neurons, but only in males, and only after the L3 stage (Yi et al., 2000; Pereira et al., 2019). However, the role of *mab-3* in these neurons is not completely mapped, and it is not known whether it is involved in regulating sexually dimorphic locomotion (Pereira et al., 2019), and loss of *mab-3* did not completely eliminate ascaroside attraction mediated by ADF (Fagan et al., 2018). Consequently, *mab-3* targets in these neurons are still unknown.

Another interesting case is the sex-shared PHC neuron class that displays highly dimorphic connectivity (Serrano-Saiz et al., 2017a). In hermaphrodites, PHC functions as a sensory neuron, presenting scarce input and output connections. Strikingly, in males, PHC receives heavy input from both male-specific and shared neurons (Jarrell et al., 2012; Cook et al., 2019). Moreover, PHC wiring changes are accompanied by morphological changes and transcriptional scaling in synaptic molecules in order to cope with the increase in the number of connections. All these changes eventually lead to the repurposing of PHC function. *dmd-3* orchestrated the entire transformation of PHC. That is, *dmd-3* is both required and sufficient to cell autonomously control the male-specific remodeling of the PHC neurons by the L4 stage (Serrano-Saiz et al., 2017a). Although it is not yet known if they are direct targets, the ectopic expression of *dmd-3* in PHC neurons in hermaphrodite animals triggered all the gene expression associated changes.

The sex-shared PHB sensory neuron forms synapses with distinct downstream interneurons in males and females, as well as to additional male-specific motor, sensory, and interneurons (Jarrell et al., 2012; Oren-Suissa et al., 2016; Cook et al., 2019). These dimorphisms in connectivity arise through two distinct mechanisms: some connections are formed only in larval males and these persist in adult males (pre-patterning for circuit wiring) while other juvenile connections between two pairs of shared-neurons (PHB>AVA and PHB>AVG) are only eliminated in one sex at L4 (selective pruning). Interestingly, both the sex of the pre-synaptic cells, PHB, and the post-synaptic site are also sufficient to initiate PHB connectivity rewiring. The dimorphic synaptic pruning events depend on the sex-specific activity of several transcription factors, including DMRTs. *dmd-5* and *dmd-10* (*dmd-10* and *dmd-11* were previously miss-annotated as two genes; now is *dmd-10*) mutants display alterations in synaptic wiring, and PHB>AVG male-specific synapses fail to be maintained in males, indicating that *dmd-5* and *dmd-10* are not required for synapse formation *per se*, but are specifically involved in controlling sex-specific synapse maintenance by preventing synaptic pruning (Oren-Suissa et al., 2016). Another *Dmrt, dmd-4* is expressed in PHA and PHB sensory neurons. In this case, *dmd-4* is maintained in hermaphrodites in these neurons, and, in *dmd-4* mutants, male specific synapses (PHB>AVG) fail to be pruned in hermaphrodites (Bayer et al., 2020). However, the precise targets of *dmd-4* which modulate synaptic connectivity are unknown.

During mating, the male engages in a precise orchestrated series of events to land on a hermaphrodite, find the vulva, and eventually inseminate the mating partner (Liu and Sternberg, 1995; Barr and Garcia, 2006). Male-specific neurons in the tail are responsible for many aspects of copulatory behavior. Therefore, it is not surprising that mutations in *Dmrt* genes, which affect the generation and specification of male-specific neurons (such as *mab-3, dmd-3* or *mab-23*), have defects in copulation (Yi et al., 2000; Lints and Emmons, 2002; Siehr et al., 2011). Mutations in *dmd* genes expressed in shared neurons also affect many facets of male mating behavior. In *dmd-5;dmd-10* double mutants, vulva location efficiency is affected as a consequence of the loss of proper connectivity between PHB and AVG. Rescue in AVG with either *dmd-5* or *dmd-10* is sufficient to rescue this behavioral defect (Oren-Suissa et al., 2016). In larval animals, the phasmid neurons PHA, PHB, and PHC function to mediate chemosensory avoidance behavior (Hilliard et al., 2002; Zou et al., 2017), a functional output which is maintained in adult hermaphrodites, but lost in adult males (Oren-Suissa et al., 2016). Similarly, the PHC neuron is important for sensing harsh touch in hermaphrodites, but not in males. Instead, male PHC is involved in mating behavior (Serrano-Saiz et al., 2017a).

In summary, *C. elegans* powerful model has allowed us to dissect how sexually dimorphic gene expression differences, how reconfiguration of morphology and connectivity rewiring of shared neurons leads to the redeployment of neurons into dimorphic behaviors, and how *Dmrts* serve as the effectors of many of these scenarios.

## *Doublesex* in Sexually Dimorphic Nervous System and Behavior in *Drosophila*

In *D. melanogaster*, sex-specific splicing of *dsx* and *fruitless* (*fru)* is the pivotal event in sexual differentiation of the nervous system. In embryonic development, the ratio of X chromosomes to autosomes causes zygotically transcribed genes to activate the RNA-splicing factor *Sex-lethal* (*Sxl*) in females only. SXL protein controls both its own splicing and the splicing of *transformer* (*tra)*, another RNA-splicing factor. Thus, TRA protein is functional only in females and causes *dsx* to be spliced, giving rise to the female-specific isoform (DSX^F^). Moreover, TRA directs a splicing in which a stop codon is introduced into female-specific *fru* mRNA, and, consequently, it is not translated. In contrast, the absence of SXL and TRA proteins in males allows the production of the male-specific isoform of DSX (DSX^M^) and FRU (FRU^M^) (Hoshijima et al., 1991; Ryner and Baker, 1991; Inoue et al., 1992; Ryner et al., 1996; Heinrichs et al., 1998; Usui-Aoki et al., 2000).

In *Drosophila*, nervous system sexual dimorphisms are related to (1) sex-specific neuronal clusters, only present in one sex, (2) sex-specific cell death and proliferation in shared particular clusters producing sex-specific numbers in homologous clusters, and (3) sex-specific neurite projections and synaptic densities. All these features are hypothesized to be responsible for sex-specific behaviors, although evidence of direct links between a given neuronal cluster and a behavioral output are sparse. Male- and female-specific DSX isoforms, together with FRU^M^, are responsible for most of those dimorphisms, executing distinct—and sometimes antagonistic—functions between the sexes to generate sex-specific neuronal traits and behaviors (Billeter et al., 2006b; Rideout et al., 2007, 2010; Kimura et al., 2008; Sanders and Arbeitman, 2008; Shirangi et al., 2009; Pan et al., 2011; Neville et al., 2014; Pan and Baker, 2014; Ishii et al., 2020).

*dsx* is expressed in the central nervous system (CNS) of larvae, pupae, and adults (Lee et al., 2002). DSX^F^ is expressed in ~700 neurons; while DSX^M^ is expressed in about 900 neurons, the majority of which also express FRU^M^ (Peng et al., 2021). The ventral nerve cord contains around two-thirds of all *dsx*-expressing neurons in the CNS, most of them found in the abdominal ganglion (Pan and Baker, 2014). In the CNS, the vast majority of *dsx*^+^ cells are cholinergic (excitatory) neurons (Zhou et al., 2014), although *dsx* expression has also been found in glial cells of both sexes (e.g., the Suboesophageal Lateral Glia, SLG, located in the anteroventral optic cleft) (Robinett et al., 2010). *dsx* is expressed in ~150 neurons per hemisphere in males, and 30–40 per hemisphere in females. In the entire adult CNS, the number of sex-specific neuronal clusters—only present in one sex—is seven in males (pC2m, pLN, pMN3, P1, SN, TN1, and TN2), and one in females (pMN2); while the number of sexually dimorphic clusters—present in both sexes but presenting different numbers—is four (pCd-1, pC1, pC2l, and abdominal ganglion) (Taylor and Truman, 1992; Nojima et al., 2021). Although, in general, the topology of *dsx*-axonal projections is similar between both sexes, male neurons notably show higher density of synapses and projections than females ones (Rideout et al., 2010). However, whether *dsx* has a direct role in the promotion of synaptic density has not yet been described. *dsx* is also expressed in the peripheral nervous system, e.g., in neuronal cells of gustatory sense organs of the male and female foreleg (Robinett et al., 2010), and in mechanosensory neurons of the male genitalia (Pavlou et al., 2016; Jois et al., 2022). Thus, in the sexually mature fly, *dsx* is expressed in key nodes of a sexually dimorphic circuitry that produces sex-specific behaviors. As we will explain in greater detail below, these nodes encompass all levels of a neural circuit: the sensory neurons that detect stimuli, the interneurons that process such stimuli, and the motor neurons that materialize the proper behavioral output.

In *Drosophila*, the main mechanisms to generate sex differences in neuron numbers involve cell proliferation and programmed cell death. Thus, DSX^M^ prolongs cell divisions of four neuroblasts in the abdominal ganglion, resulting in about 20 extra neurons in adult males compared to females (Taylor and Truman, 1992). Furthermore, these terminal neuroblasts do not develop in *dsx* null flies (Taylor and Truman, 1992), which is consistent with the decreased number of male-specific serotonergic neurons in the abdominal ganglion of *dsx* null males (Billeter et al., 2006a). These serotonergic neurons control seminal fluid and sperm transfer (Lee and Hall, 2001; Lee et al., 2001), are patterned in two clusters (dorsal and ventral), and most of them co-express FRU^M^ and DSX^M^ (Billeter et al., 2006a). In the posterior part of the protocerebrum, DSX^M^ also promotes additional neuroblast divisions in males, promoting a higher number of neurons in males in two *dsx*^+^ neuronal clusters (pC1 and pC2) (Sanders and Arbeitman, 2008; Rideout et al., 2010; Robinett et al., 2010). Likewise, DSX^F^ determines female-specific neuronal numbers configuring the female-specific circuitry. Several studies show that DSX^F^ promotes cell death in different contexts: in sex-specific neuroblasts of the terminal abdominal neuromeres (Birkholz et al., 2013), in the pupal and adult TN1 cluster of the thoracic ventral nerve cord (Sanders and Arbeitman, 2008), and in P1 interneurons of the adult brain (Kimura et al., 2008). TN1 cluster—which disappears completely in females by 48 hours after pupal formation—is a good scenario to illustrate the antagonistic actions of the sex-specific DSX isoforms. While DSX^F^ is necessary for female-specific apoptosis in TN1 progenitors, and no TN1 neurons are generated in females, DSX^M^ promotes additional cell divisions to achieve around 15 ± 0.5 TN1 cell neurons. In fact, XX mutant flies expressing both DSX^M^ and DSX^F^ show an intermediate number of TN1 cells (5 ± 0.3 cells) (Sanders and Arbeitman, 2008). Additionally, DSX also has a role in generating sexual dimorphisms in neuronal processes. In cooperation with FRU, DSX establishes male-specific midline crossing of axons from foreleg gustatory receptor neurons (GRN). In particular, DSX^F^ represses this midline crossing, while DSX^M^ might be promoting it (Mellert et al., 2010). Also, *dsx*^+^ aDN neurons display sexually dimorphic arborizations patterns, suggesting that males and females receive information from distinct sensory modalities (Nojima et al., 2021).

Evidence that *dsx* regulates sexual neuronal circuit differentiation comes initially from behavioral analysis of *dsx* mutant males and females. Male courtship in *D. melanogaster* is one of the best-studied animal behaviors (Hall, 1994; Yamamoto and Koganezawa, 2013), although aggression, virgin female receptivity, and female post-mating behaviors in flies are also interesting. All these mentioned behaviors are either sex-specific (e.g., male courtship) or sexually dimorphic (e.g., aggression), and they depend on *dsx*-expressing neurons (Pan et al., 2011; Kimura et al., 2015; Koganezawa et al., 2016). Since the first evidence of the atypical sexual behavior—performing and eliciting courtship—of XY homozygous mutants for *dsx* was described in 1985 (McRobert and Tompkins, 1985), a lot of research has been done to better understand the role of *dsx* in the control of sexual behavior. Courtship consists of a stereotyped sequence of actions displayed by the male to interest the female in copulation. Despite the fact that copulation in *dsx* null mutants is physically impossible because they do not have external genitalia (Taylor et al., 1994), *dsx* XY flies can perform, in a qualitatively normal-appearing manner, the courtship steps of following/orientation, tapping, wing extension, licking, and copulation attempts (Villella and Hall, 1996). However, *dsx* XY mutants exhibit a reduced wing extension time, and some individuals do not perform tapping, licking, or copulation attempts at all, with a high variability in phenotype penetrance (Villella and Hall, 1996). More recent studies demonstrated that the *dsx* XY fly courtship phenotype is a consequence of defects in the male motor circuit for copulation located in the abdominal ganglion (Pavlou et al., 2016). XX flies expressing constitutively DSX^M^ do not exhibit courtship toward wild-type females (Taylor et al., 1994), suggesting that *dsx* alone cannot specify courtship behavior, and other regulators such as the *fru* gene are needed (Rideout et al., 2007; Yamamoto and Koganezawa, 2013).

On the other hand, *dsx* is fully required for proper courtship singing in males. The courtship song is crucial to stimulate the female to mate, consisting of brief pulses (*pulse song*) and bouts of humming sound (*sine song*) (Griffith et al., 1993; Rybak et al., 2002). *dsx* XY mutants are unable to sing *sine song*, and they present defects in particular aspects of *pulse song* (Villella and Hall, 1996; Shirangi et al., 2016). The complete absence of *sine song* in these mutants is due to the function of *dsx* in regulating the connectivity between the TN1A interneurons, a subclass of TN1 interneuron, and hg1 motor neurons (Shirangi et al., 2016). The activation or inhibition of some (or all) of the *dsx*-expressing neurons dramatically alters male courtship and copulatory behaviors. In males, the activation of *dsx*-expressing neurons triggers all courtship and copulatory behaviors (Pan et al., 2011), while their inhibition blocks them (Rideout et al., 2010).

*dsx* not only controls the male ability to perform the courtship ritual, but also the capacity of both males and females to elicit courtship in conspecifics. Thus, *dsx* XY flies elicit courtship in wild-type males at abnormally high levels. Interestingly, *dsx*^*F*^ transgene-expressing males—XY flies ectopically expressing the female isoform of *dsx*—are strongly courted by normal wild-type males and other *dsx*^*F*^ transgene males. Presumably, the sex appeal acquired by *dsx*^*F*^ transgene-expressing males is due to the concomitant feminization of their synthesis of pheromones (Waterbury et al., 1999). Accordingly, DSX^F^—but not DSX^M^—activates the *desaturase-F* gene, which encodes the enzyme involved in the synthesis of dienes pheromones, crucial for optimal male courtship behavior (Chertemps et al., 2006; Shirangi et al., 2009).

Over the course of the last decade, insights into the function of DSX^F^ in female sexual behavior have finally been investigated. *dsx*-positive neurons are critical for receptivity to first copulation (pC1 and pCd) in virgin females, for post-mating behaviors such as sperm ejection and storage (Dh44R1 neurons), for oviposition (mechanosensory neurons of the female reproductive system, abdominal ganglion, pMN2, and aDN), and for rejection behavior toward courting males (pC2l, abdominal ganglion) (Rezával et al., 2012, 2014; Zhou et al., 2014; Kimura et al., 2015; Lee et al., 2015; Nojima et al., 2021).

Lastly, competition over territory, food, or mates triggers aggression behavior between pairs of male or female flies (Kravitz and Huber, 2003). It has been demonstrated that the *dsx*-positive pC1 cluster resolves the males' dilemma between fighting or courting when they encounter conspecific. Thus, pC1 *fru*-negative sub-fraction acts as an aggression-triggering center, whereas pC1 *fru*-positive sub-fraction acts as a courtship-triggering center, and these two centers are mutually exclusively activated by two *fru*-single positive clusters, LC1 and mAL (Koganezawa et al., 2016). Furthermore, the activation of a subset of *dsx*-expressing pC1 neurons increases aggression behavior in females, but not in males (Palavicino-Maggio et al., 2019). Therefore, once again, we have evidence of *dsx*-expressing neurons contributing to control a sexually biased behavior.

## *Dmrt* Gene Expression and Function in the Nervous System of Diverse Metazoan Groups, Including Humans

Once we comprehended how *Dmrt* genes control sex differentiation of the nervous system in *C. elegans* and *D. melanogaster*, we sought to investigate how prevalent *Dmrt* genes are in nervous systems across Metazoa, with special attention to their potential role in sexual differentiation. Unfortunately, the majority of these studies do not systematically examine both sexes, and in the few cases where *Dmrt* expression is compared between the sexes, quantitative approaches or enough anatomical resolution are lacking, so dimorphic expressions cannot be unraveled.

Most metazoan studies on *Dmrt* expression in the nervous system have been conducted in chordates, and among them, studies in fishes stand out for including 11 distinct species. However, studies in DMRT neuronal function have been exclusively focused on model organisms: rat, zebrafish, and *Xenopus*, but primarily in mouse. For example, in these four species, members of the DMA subfamily (*Dmrt3, Dmrt4*, and *Dmrt5*) have been shown to have a conserved role in neurogenesis (Huang et al., 2005; Yoshizawa et al., 2011; Konno et al., 2012, 2019; Kikkawa et al., 2013; Parlier et al., 2013; Saulnier et al., 2013). For excellent reviews see (Bellefroid et al., 2013; Kikkawa and Osumi, 2021).

According to our literature research (summarized in Table 1), *Dmrt* genes are expressed in neural tissues in a plethora of animal species, including the phyla Chordata, Arthropoda, Platyhelmithes, Onychophora, and Cnidaria. Many of the studies do not reference the sex of the samples analyzed [we indicate this in the table with a (–) in the *Dimorphic expression* column], or only one sex was analyzed (also indicated in the table). Only 12 of the 53 studies included sex comparisons, and almost all of them deal with fishes or arthropods (Japanese pufferfish, largemouth bass, Medaka, tilapia, platyfish, Atlantic cod, swamp eel, sweetfish, mosquito, bumblebee, silkmoth, and oriental river prawn). Sex differences in *Dmrt* expression levels or expression patterns have been reported in six studies (highlighted in color in Table 1): sex differences in mRNA levels for *dmrt4* were found in the adult telencephalons of tilapia (Cao et al., 2007), and in the adult brains of Atlantic cod (Johnsen and Andersen, 2012); for *dmrt5*, in the adult brains of the sweetfish (Wang et al., 2014); for *dsx*, in the adult brains of the silkmoth (Nakata et al., 2021); and for *dmrt11E*, in the adult abdominal ganglia of the oriental river prawn (Wang et al., 2019). Regarding sex differences in expression patterns, *dsx* is expressed in different clusters in the antennal lobe and mushroom body of the mosquito *Aedes aegypti* according to sex (Tomchaney et al., 2014). In the silkmoth, besides the sex differences in *dsx* expression across development, *dsx* is expressed ventral to the calyx of the mushroom body (cluster 1) and dorsolateral to the esophagus (cluster 2) in males, while in the female *dsx* is only expressed in cluster 1 (Nakata et al., 2021).

To investigate sex differences in *Dmrt* expression, the major limitation of the above mRNA quantifications is their lack of cell resolution. In many studies, whole telencephalon or brain was analyzed, or the authors did not specify whether they dissected a particular region. However, by taking the whole tissue, putative sex differences in particular brain nuclei or cell subpopulations are neglected. In fact, that could be occurring with the similar expression of *dmrt1* and *dmrt5* observed in the adult brains of males and females in the largemouth bass (Yan et al., 2019) and Atlantic cod (Johnsen and Andersen, 2012), respectively. Interestingly, although not discussed in the article, during gonad transformation in the swamp eel (this animal undergoes sex reversal; first, they have ovaries, then there is an intersex stage between female and male stages in which the animal develops ovotestes, and finally, they develop testes) expression of *dmrt2, dmrt2b, dmrt3, dmrt4*, and *dmrt5* in the brain undergoes concomitant changes, as observed in the differential intensity of bands in cDNA electrophoretic gels (Sheng et al., 2014). There is another study where an evident sex differential expression in the Japanese pufferfish brain for a *dmrt* exists, but the authors do not mention it (Yamaguchi et al., 2006). Although a quantitative approach was not used, a higher expression of *dmrt5* in females can be observed by RT-PCR followed by Southern blot.

Concerning humans, according to the BrainSpan Atlas of the Developing Human Brain (http://www.brainspan.org/), a transcriptome resource of prenatal and post-natal human specimens of cortical and subcortical structures, *DMRT2, DMRTA1/DMRT4, DMRT3* and *DMRTA2/DMRT5* are expressed mainly in cortical regions and at higher levels in prenatal stages. In addition to the expression data, two clinical case reports have served to provide insights into how *DMRTs* operate in the human nervous system. One case report suggests that loss of function of *DMRTA2* leads to a severe recessive condition characterized by microcephaly, lissencephaly, and agenesis of the corpus callosum (Urquhart et al., 2016). In fact, *Dmrt5*^−/−^ mice, among other defects, show a strong reduction of the caudomedial cortex, and they lack corpus callosum (Saulnier et al., 2013), which is coherent with the observed human phenotype. On the other hand, Lerer et al. (2005) reported a case of a familial cerebral palsy with a deletion in the *KANK1* gene locus, including a large part of the intergenic region between *KANK1* and *DMRT1* (*KANK1-DMRT1-DMRT3* constitute a synteny in both humans and mice) (Lerer et al., 2005; Kubota et al., 2018). However, this clinical case report does not directly elucidate the role of *DMRT3* in cerebral palsy, but it served as a basis for a subsequent study that demonstrates the involvement of *DMRT3 via* bioinformatics approaches (Kubota et al., 2018). In that study, the authors used comparative genomic and reanalysis of public data for ChIP-Seq and chromatin interaction to postulate an enhancer of *DMRT3* as a putative source for spastic cerebral palsy. In addition, this presumed enhancer contains a putative RAR/RXR-binding motif, and *Dmrt3* gene expression is upregulated after retinoic acid stimulation in mouse cell culture (Kubota et al., 2018). Since *Dmrt3* participates in the specification of spinal cord dI6 interneurons [mutations in the *Dmrt3* gene affects locomotion in horses and mice (Andersson et al., 2012)], and *dmrt3* is involved in cortical development (Konno et al., 2019), if conserved in these features, *DMRT3* could contribute to motor symptoms in cerebral palsy patients as a consequence of impaired development of the motor cortex and dysfunctional locomotion circuits in the spinal cord. Lastly, deletion or duplication of the short arm of chromosome 9, where *DMRT1, DMRT2*, and *DMRT3* are located, is characterized by developmental delay, intellectual disabilities, and craniofacial anomalies (Güneş et al., 2017). In addition, an association of 9p deletion and autistic spectrum disorder has also been reported (Õunap et al., 2004; Vinci et al., 2007; Yang et al., 2012).

One limitation of our search is that we have not systematically looked for neural tissues in animals in which it was demonstrated that *Dmrt* are not expressed, which would provide more information about *Dmrt* conservation across the animal kingdom. For instance, some *Dmrts* are not detected in the adult brains of males and females of several fishes: *dmrt6* in rock bream (supplementary material in Li et al., 2021), *dmrt4* in Japanese pufferfish (Yamaguchi et al., 2006), *dmrt1, dmrt2a*, and *dmrt3* in Atlantic cod (Johnsen and Andersen, 2012), and *dmrt1* in sweetfish (Wang et al., 2014).

## *Dmrt* Genes Non-Cell Autonomous Effects in the Brain

As we have exposed previously, the role of *Dmrt* genes in sexual differentiation and regulation of vertebrate's nervous system has not been systematically approached. However, we would like to delve into potential non-cell autonomous interactions between *Dmrt* functions in peripheral organs and the brain. In mammals, sexual hormones secreted by the gonad have a fundamental non-cell autonomous role in the nervous system. In the embryo, the coordinated action of fetal Leydig and Sertoli cells is necessary for testosterone release (Shima et al., 2013). Embryonic testosterone is subsequently locally converted to estradiol by aromatase to act on brain cells. According to the organizational-activational hypothesis of brain sexual differentiation (Arnold, 2009), during the *organizational* phase, several mechanisms will configure dimorphic circuits, such as proliferation, neurogenesis, apoptosis, dendritic growth, synaptogenesis or synaptic pruning among others (Phoenix et al., 1959; Arnold, 2009; McCarthy et al., 2017). Later, in sexually mature adult animals, both testosterone and estradiol (released by adult Leydig cells and by granulosa cells, respectively) will modulate brain activity during the *activational* phase (McCarthy, 2020; Gegenhuber et al., 2022). Therefore, transcriptional networks—potentially including *Dmrt* genes—that affect gonadal development and maintenance could indirectly have a major impact on sexual brain differentiation.

Despite profound differences in gonad structure and development among animal phyla, *Dmrt* genes are specifically expressed in the developing gonads of almost all animals (Matson and Zarkower, 2012). In mice and humans, *Dmrt1* expression is sex-specific and developmentally dynamic. In mice (where it has been most extensively studied), *Dmrt*1 is expressed in germ cells of both sexes through E13.5, disappearing by about E15.5 (Lei et al., 2007). However, *Dmrt1* becomes highly male-enriched in somatic Sertoli cells by about E14.5 as testis differentiation initiates, and its expression is maintained thereafter (Raymond et al., 1999; Lei et al., 2007). In males, *Dmrt1* is re-expressed in spermatogonia just before birth and maintained in spermatogonia subsequently (Lei et al., 2007; Matson et al., 2010). Human *DMRT1* expression is less well-characterized but appears to be similar to that of mice. During fetal development, *DMRT1* mRNA is detectable by gestational week 11 (GW11) in both sexes, becoming most abundant in pre-Sertoli cells between GW10 and GW20 (Jorgensen et al., 2012). In adult testes, *DMRT1* is expressed both in Sertoli cells and in spermatogonia, as in mice. However, there are discrepancies between the functions ascribed to *DMRT1* in humans and mice that are not fully understood yet. In *Dmrt1* mutant mice, severe defects are observed in post-natal testes and reveal the importance of *Dmrt1* in Sertoli differentiation and male germ cell survival. About two weeks after birth, *Dmrt1* mutant immature Sertoli cells acquire expression of female-specific granulosa cell markers (Matson et al., 2011). Interestingly, androgen activity is reduced and estradiol levels are elevated in mutants, suggesting a male-to-female tilt in hormone signaling, which is probably due in part to an elevated expression of the enzyme aromatase. However, as far as we are aware, nervous system sexual differentiation has not been assessed in these mice. Human *DMRT1* deletions and microdeletions on the distal part of chromosome 9, where *DMRT1* is located, and missense mutations in the DM domain have been associated with Disorders in Sexual Development (DSD), and reported to cause partial or complete 46,XY gonadal dysgenesis (Raymond, 1999; Zarkower and Murphy, 2021) and male infertility cases (Tewes et al., 2014). The degree of DSD can range from male genitalia with small dysgenic testes, to complete sex reversal that will potentially affect testosterone secretion. Moreover, distal 9p monosomy deletions have also been associated with cognitive deficits, developmental delay, and a characteristic array of dysmorphic craniofacial features (Alfi et al., 1976). 9p deletions consistently affect *DMRT1*. However, most of these deletions also affect other genes, including the adjacent paralogues *DMRT3* and *DMRT2*, which are immediately proximal to *DMRT1* on 9p. In mice, *Dmrt3* function in gonadal development (and the nearby *Dmrt2*) has not been reported (Seo et al., 2006; De Clercq et al., 2016; Desmaris et al., 2018).

In vertebrates, most *Dmrt* family members are expressed in the undifferentiated gonad and in most cases, they are subsequently maintained at higher levels in male as opposed to female gonads (Kim et al., 2003). In mice, at least, mutations in *Dmrt4* (*Dmrta1*), *Dmrt6* (*Dmrtb1*), and *Dmrt7* (*Dmrtc2*) disrupt gonadal development in one or both sexes (Raymond et al., 2000; Balciuniene et al., 2006; Kawamata and Nishimori, 2006; Kim et al., 2007; Zhang et al., 2014). However, these *Dmrt* genes are expressed in cells that do not affect gonadal hormone secretion. *Dmrt7* localizes to spermatocytes (Kawamata and Nishimori, 2006); *Dmrt6* and *Dmrt7* regulate germ cell differentiation and appear not to be required in any other cell types (Kim et al., 2007; Zhang et al., 2014). A systematic analysis of *Dmrt* expression in embryonic mouse gonads by RT-PCR revealed that many of them are expressed in male and female gonads at some point of development (Kim et al., 2003). *Dmrt3* is expressed in embryonic ovaries and testes, but is undetectable in adults. Conversely, *Dmrt4* and *Dmrt5* are expressed in adult testes. In the embryo, *Dmrt5* is more highly expressed in the ovary, although no gonadal phenotype in *Dmrt5* mutants has been described to date, and *Dmrt5* null mutants are perinatally lethal and fertility assays have not yet been performed. *Dmrt4* is present in the gonads of both embryos and adult animals (Kim et al., 2003; Balciuniene et al., 2006) and while mutant females have polyovular follicles, suggesting a role in folliculogenesis, 25% of mutant males consistently exhibited copulatory behavior toward other males (Balciuniene et al., 2006). However, the origin of this behavior has not yet been fully explored molecularly.

Another interesting site of *Dmrt* expression that we would like to briefly highlight, is the potential role of *Dmrt5* in pituitary gland development. The hypothalamic-pituitary-gonadal axis controls puberty and reproduction (Abreu and Kaiser, 2016). This axis is active in the embryonic and early post-natal stages of life, is subsequently restrained during childhood, and its reactivation culminates in puberty initiation. At that time, the hypothalamus releases gonadotrophin-releasing hormone (GnRH), which can act on pituitary gonadotropes, triggering the release of follicle-stimulating hormone (FSH) and luteinizing hormone (LH). FSH and LH bind to their respective receptors in the ovary, leading to secretion of the sex steroid hormones, estrogen, which stimulates vitellogenesis, and progesterone, which stimulates oocyte meiosis, follicular maturation, and ovulation, even in fish (Yaron and Levavi-Sivan, 2011). Interestingly, in zebrafish pituitaries, *dmrt5* controls corticotrope and gonadotrope cell differentiation (Graf et al., 2015). It is tempting to speculate that altered *dmrt5* activity could indirectly affect gonadal activity and consequently sexual development because of altered gonadotrope numbers and/or activities. Although this has not been tested, a recent study reported that a deficiency in LH and FSH in zebrafish mutants leads to a significant delay in gonad development and the onset of puberty, as well as infertility and sex reversal (Zhang et al., 2015). Interestingly, *Dmrt5* is expressed in the embryonic mouse pituitary (Saulnier et al., 2013), and according to *The Human Protein Atlas* resource, *DMRT5* (*DMRTA2)* is enriched in pituitary and testes tissues (and not in ovaries) (www.proteinatlas.org).

## Conclusions and Future Perspectives

Regardless of the independent evolutionary origins of *Dmrt1, dsx*, and *mab-3* (Mawaribuchi et al., 2019), research within the last couple of decades has positioned DMRT transcription factors as the most conserved type of protein consistently utilized throughout the animal kingdom to control sex determination and differentiation. We have extensively detailed how *Dmrt* genes operate in the sexual differentiation of the nervous systems in *C. elegans* and *Drosophila*. In light of the similarities between mechanistic aspects of *dsx* and *dmds*, it is possible that *Dmrt* genes control similar target genes to cooperate in the sexual differentiation of the brain in the nervous system of other animals. Indeed, *dsx* and *Dmrt1* have been proposed to regulate similar target genes (Clough et al., 2014). Neurogenesis, cell death, and connectivity—through the control of morphology, neurotransmitter gene expression, or selective synaptic pruning—seem to be the preferred features controlled by *Dmrt* genes that, ultimately, lead to the generation of sexually dimorphic circuits and behavioral outputs. Significantly, these are recurrent mechanisms that are engaged in the refinement of neuronal circuits at various stages throughout development. It is precisely the fact that *Dmrt* genes control such mechanisms that situates them in a relevant position in the control of sexually dimorphic circuit configurations.

In all organisms studied so far, the precise role of *Dmrts* in the specification of sex-specific somatic features, including in the nervous system, results from a dichotomy in their expression pattern in males and females. Up-stream sex-determinant primary cues ensure the sexually dimorphic expression of *Dmrt* genes: for *dsx*, a single locus produces two different variants, a female and a male variant deployed in many different cellular contexts; in *C. elegans*, several *dmds* are expressed exclusively in males (and *dmd-4* in hermaphrodites) as a result of the actions of the master regulator of sex-determination *tra-1*. In other species, where *Dmrts* are dimorphically expressed, upstream direct regulators have not yet been identified. However, as we have exposed in this review, not all *Dmrt* genes are dimorphically expressed (Table 1). One possibility is that the consequences of this loss of dimorphic expression could lead to unisex functions (Bellefroid et al., 2013). However, we hypothesize that as a consequence of the monomorphic expression, in certain cellular contexts, *Dmrt* genes could have acquired a *neofunction* to prevent sexual differences from occurring. However, to date, no functional studies have been performed in *Dmrt* mutants to demonstrate differential outcomes in males and females in *Dmrt* unisex-expressing regions.

**Figure 2:**
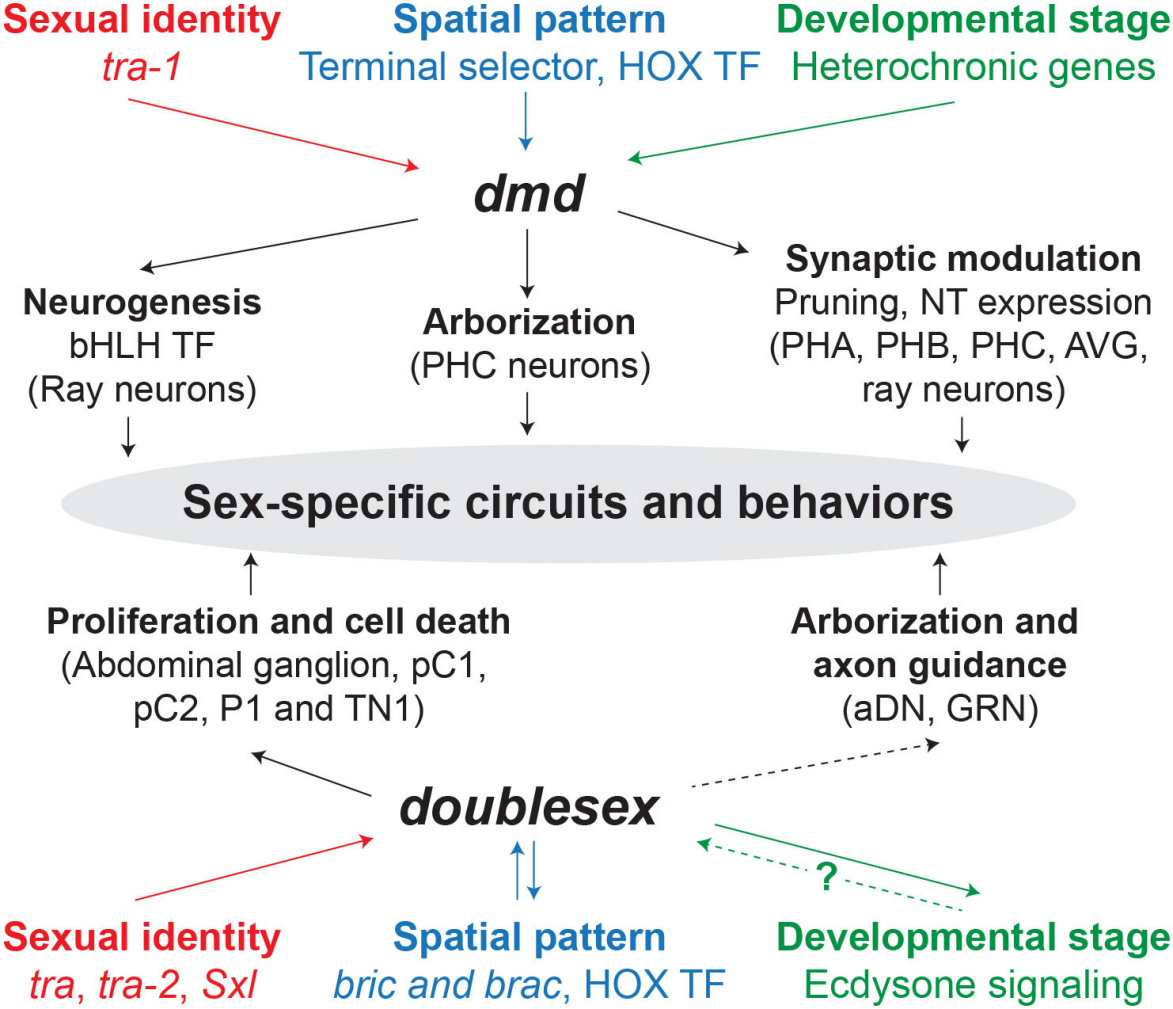
*dmd* and *doublesex* mechanisms underlying sex-specific circuits and behaviors. Solid and dashed arrows indicate direct and indirect regulation, respectively. The question mark represents that the mechanism by which ecdysone signaling regulates *dsx* is unknown. TF, transcription factor; NT, neurotransmitter.

The relatively simple and fully mapped nervous systems of *C. elegans* and *Drosophila* in both sexes (in *Drosophila*, the female neuronal connectivity map has been made available recently, and the male map will soon be released), together with their genetic amenability and access to sexually dimorphic behaviors, has allowed the thorough delineation of the role of *Dmrts* in sexual differentiation in these animals. In other species, especially in vertebrates, this task might entail a greater challenge due to the greater complexity of their nervous systems and the lack of powerful genetic tools. However, in order to advance our knowledge of the potential functions of *Dmrt* genes across metazoans (with special focus on the mammalian brain), we would like to highlight a few specific remarks:

- Many of the recent/contemporary studies relied on *Dmrt* mRNA measurements (either RT-PCR or *in situ* hybridization). However, by relying solely on these techniques, post-translational regulatory mechanisms will go undetected. This point is especially relevant in *Dmrt* genes with DMA domain that may undergo sex-specific post-transcriptional regulation, as has been proposed in Bayer et al. (2020).
- *Dmrt* genes may be acting in discrete, scattered, neuronal subpopulations. Within neuronal circuits, subtle changes in synaptic connection affect the communication capabilities of neurons (for instance, by scaling their neurotransmitter levels up or down, or switching post-synaptic partners) and could have major behavioral outcomes. Indeed, this is in line with the notion that homologous circuits between males and females can be reversibly modulated. Searching for neuron-type specific *Dmrt* expression differences requires the use of technical approaches with high cellular resolution and quantitative characteristics. In this regard, differences in *Dmrt* level of expression could also be highly relevant.
- If *Dmrt* genes are expressed in a single neuron type within the circuit, the mutant phenotype may have major behavioral consequences. Therefore, functional studies, based on mutant strains, would be a useful and necessary complement to *Dmrt* gene expression maps.
- *Dmrt* genes act as integrators of sex, space, and time (Figure 2). However, the precise molecular mechanisms to integrate these variables are not yet fully understood and still require additional investigation, even in the most-studied invertebrate model organisms. Therefore, when studying *Dmrt* expression and function, different time points should be considered. In some cases, embryos and adult—sexually mature—animals have been analyzed, but usually a systematic approach throughout the various developmental stages is lacking. For instance, in mammalian brains, *Dmrt* gene function might be more relevant during the embryonic testosterone surge or during/after puberty. Lastly, many mouse *Dmrt* mutants are embryonic lethal and have not yet been studied during post-natal stages, when sexual behaviors emerge.
- Lastly, we would like to emphasize that *Dmrt* functions should be studied using a holistic approach. *Dmrt* genes are widely expressed in many organs. It may be especially interesting to explore the non-cell autonomous relationships that *Dmrt* phenotypes in the gonad or the pituitary gland might exert on brain differentiation.

From a human health perspective, *DMRTs* are excellent genes to be considered as candidate factors related to the etiology and sexual skewing of neuropsychiatric diseases. In the mammalian brain, where sex hormones have a huge impact in establishing sex differences (McCarthy and Arnold, 2011), DMRTs may be acting in parallel in order to create or buffer differences created by gonadal hormones. Hence, *DMRTs* may either afford protection or generate vulnerability in one sex versus the other for sex-biased mental disorders such as autism spectrum disorder, bipolar disorder, and schizophrenia.

Moreover, given the importance of sex in this review, we feel obliged to remark, regrettably, that most neuroscience research is conducted in males, and even when both sexes are included, the sex is rarely analyzed as a variable (Beery and Zucker, 2011). We hope that this review also serves to raise awareness within the scientific community to more frequently consider sex as a biological variable.

## Author Contributions

RC-N and ES-S wrote the text. Both authors contributed to the article and approved the submitted version.

## Funding

This work was supported by Grant PGC2018-101751-A-100, by MCIN/ AEI /10.13039/501100011033/ and by ERDF “A way of making Europe”. RC-N was supported by Ministerio de Universidades - FPU19/02352. ES-S was supported by Grant RYC-2016-20537 funded by MCIN/AEI /10.13039/501100011033 and ESF “Investing in your future”.

## Conflict of Interest

The authors declare that the research was conducted in the absence of any commercial or financial relationships that could be construed as a potential conflict of interest.

## Publisher's Note

All claims expressed in this article are solely those of the authors and do not necessarily represent those of their affiliated organizations, or those of the publisher, the editors and the reviewers. Any product that may be evaluated in this article, or claim that may be made by its manufacturer, is not guaranteed or endorsed by the publisher.
